# Correction: Sauger et al. A Quantitative Histologic Analysis of Oogenesis in the Flatfish Species *Pleuronectes platessa* as a Tool for Fisheries Management. *Animals* 2023, *13*, 2506

**DOI:** 10.3390/ani14233409

**Published:** 2024-11-26

**Authors:** Carine Sauger, Jérôme Quinquis, Clothilde Berthelin, Mélanie Lepoittevin, Nicolas Elie, Laurent Dubroca, Kristell Kellner

**Affiliations:** 1 Unity Biology of Organisms and Aquatic Ecosystems (UMR 8067 BOREA), University of Caen-Normandie, Muséum National d’Histoire Naturelle, Sorbonne University, CNRS, IRD, Université des Antilles, Esplanade de la Paix, 14032 Caen, France; clothilde.berthelin@unicaen.fr (C.B.); melanie.lepoittevin@unicaen.fr (M.L.); 2 Laboratoire Ressources Halieutiques de Port en Bessin, Institut Français de Recherche pour l’Exploitation de la Mer (IFREMER), Avenue du Général de Gaulle, 14520 Port en Bessin Huppain, France; 3 Service Unit PLATON, VIRTUAL’HIS, Federative Structure 4207 “Normandie Oncologie”, Normandie University UNICAEN, 14000 Caen, France; nicolas.elie@unicaen.fr

Error in the Figure.

In the original publication [1], there was a mistake in “Figure 9” as published. After a review of the GSI calculations, Figure 9a was updated, and the text was modified accordingly. These corrections were shown in the final template sent. However, in the final print, changes to the text were made, but Figure 9 was not changed. This led to Figure 9a not showing the correct values but instead displaying the old values (before the new calculation method). The corrected Figure 9 appears below.

## Figures and Tables

**Figure 9:**
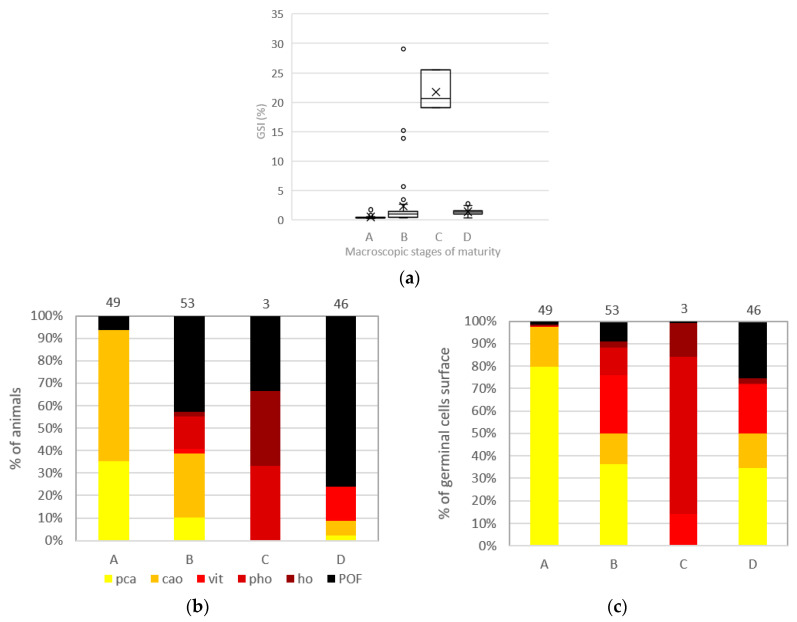
*Pleuronectes platessa* classified under a maturity phase through visual macroscopic criteria with (**a**) boxplot of gonadosomatic index (GSI %) with minimum, first quartile, median, third quartile, maximum. Black cross indicates the mean value. (**b**) percentage of animals by the most advanced oocyte stage found within their ovary and (**c**) mean % GCS (germline cell surface). With A: immature; B: developing; C: spawning; D: regressing/regenerating. Germline cells were identified through stereology and the percentages were computed from the total germ cell population in each maturity phase. pca: precortical alveoli oocytes (og+po1+po2); cao: cortical alveoli oocyte; vit: vitellogenic oocyte; pho: partially hydrated oocyte; ho: hydrated oocyte; POF: post-ovulatory follicle. The number of fish sampled for each month (n) is indicated on the top of each bar for (**b**,**c**).
